# The Impact of Clinical Factors, Vitamin B12 and Total Cholesterol on Severity of Anorexia Nervosa: A Multicentric Cross-Sectional Study

**DOI:** 10.3390/nu15234954

**Published:** 2023-11-29

**Authors:** Letizia Maria Affaticati, Massimiliano Buoli, Nadia Vaccaro, Francesca Manzo, Alberto Scalia, Sara Coloccini, Tommaso Zuliani, Davide La Tegola, Enrico Capuzzi, Monica Nicastro, Fabrizia Colmegna, Massimo Clerici, Antonios Dakanalis, Alice Caldiroli

**Affiliations:** 1 Department of Medicine and Surgery, University of Milan Bicocca, Via Cadore 38, 20900 Monza, MB, Italy; letizia.affaticati@gmail.com (L.M.A.); n.vaccaro@campus.unimib.it (N.V.); f.manzo5@campus.unimib.it (F.M.); a.scalia5@campus.unimib.it (A.S.); massimo.clerici@unimib.it (M.C.); 2 Department of Neurosciences and Mental Health, Fondazione IRCCS Ca’Granda Ospedale Maggiore Policlinico, 20122 Milan, MI, Italy; massimiliano.buoli@unimi.it; 3 Department of Pathophysiology and Transplantation, University of Milan, 20122 Milan, MI, Italy; 4 Department of Clinical and Experimental Medicine, Institute of Psychiatry, University of Catania, 95124 Catania, CT, Italy; sara.coloccini@gmail.com; 5 Department of Medicine and Surgery, University of Milan, 20122 Milan, MI, Italy; tommaso.zuliani@studenti.unimi.it; 6 Department of Mental Health, Fondazione IRCCS San Gerardo dei Tintori, Via G.B. Pergolesi 33, 20900 Monza, MB, Italy; davide.lategola@irccs-sangerardo.it (D.L.T.); e.capuzzi1@campus.unimib.it (E.C.); monica.nicastro@irccs-sangerardo.it (M.N.); fabrizia.colmegna@irccs-sangerardo.it (F.C.); alice.caldiroli@irccs-sangerardo.it (A.C.)

**Keywords:** anorexia, severity of illness, body mass index (BMI), nutrition, vitamins, peripheral biomarkers, eating disorders

## Abstract

Severe forms of Anorexia Nervosa (AN) are characterized by medical complications, psychiatric comorbidity, and high mortality. This study investigated potential associations between clinical/biological factors and the severity of AN, measured by the Body Mass Index (BMI). Red and white blood cells, hemoglobin, platelets, iron, vitamins D and B12, folate, and total cholesterol were measured in a mixed sample of 78 inpatients and outpatients. Linear regressions and one-way analyses of variance (ANOVAs) were carried out to evaluate the relationship between BMI and clinical/biochemical variables. BMI was significantly lower in hospitalized patients (F = 4.662; *p* = 0.034) and in those under pharmacological treatment (F = 5.733; *p* = 0.019) or poly-therapy (F = 5.635; *p* = 0.021). Higher vitamin B12 (β = −0.556, *p* < 0.001), total cholesterol (β = −0.320, *p* = 0.027), and later age at onset (with a trend towards significance) (β = −0.376, *p* = 0.058) were associated with a lower BMI. Increased total cholesterol and vitamin B12, later age at onset, current pharmacological treatment, and poly-therapy might be distinctive in patients with a lower BMI. In clinical practice, these findings may contribute to the early identification of AN patients at higher risk of developing complicated or chronic forms of the disorder. Further studies on larger samples are needed to identify potential predictive factors of AN severity in the framework of precision medicine.

## 1. Introduction

Anorexia nervosa (AN) is a serious mental disorder characterized by an intense fear of weight gain, resulting in persistent restriction of dietary intake and low body weight [1]. Although AN can affect all individuals, it is most common among young women, with lifetime prevalence rates up to 6.3% [2]. AN is associated with a high risk of chronic course and is frequently comorbid with other psychiatric and medical conditions, especially in its most severe forms [3,4,5]. Estimated mortality rates are the highest among all mental disorders, with deaths occurring as a result of medical sequelae or suicide [6,7].

With dietary restriction a core symptom of the disorder, individuals with AN can present several nutritional alterations. Depending on the degree of malnourishment, micronutrient deficiencies are detected in a range of 45.9–92.8% of patients and include zinc, vitamin D, copper, selenium, vitamin B1, vitamin B12, and vitamin B9 deficits [8,9,10]. Despite some contradictory findings [11,12], nutritional parameters may represent biomarkers of the presence and course of the disorder. Metabolic changes are also a potential area of interest in the identification of AN biomarkers [13,14]. Among them, higher total cholesterol (TC) levels compared to healthy controls have repeatedly been identified [15].

Although most studies have explored potential biomarkers in comparison to healthy individuals, to date, there are no established markers for the different degrees of AN severity [16]. Among micronutrients, only selenium, vitamin B12, and folic acid have been investigated as putative indicators of AN severity. Specifically, plasma levels of vitamin B12 and folate seem to increase proportionally with the degree of severity [11,17], while selenium deficiency might characterize subjects with more severe AN [10]. Interestingly, a recent study reported higher cholesterol levels in subjects with a more critical course of the disorder [18]. However, to our knowledge, no studies have delved into the identification of metabolic alterations as markers of AN severity per se rather than starvation status.

Among the various hematological and immunological abnormalities documented in AN [19,20,21,22], bone marrow degeneration has been linked to the severity of weight loss [23], while concentrations of the chemokine CCL11 seem to be inversely related to Body Mass Index (BMI) [24]. Furthermore, the neutrophil-to-lymphocyte ratio (NLR) and platelet-to-lymphocyte ratio (PLR) showed significant correlations with lower BMI [17] and alterations in bone mineral density [25]. Changes in bone mineral density have also been associated with leptin and adiponectin levels [26]. Notably, the ratio of high molecular weight adiponectin (i.e., the active form of the protein) to total adiponectin seems to be higher in underweight AN patients with osteoporosis compared to recovered individuals [26].

Aside from biochemical alterations, a correlation between olfactory sensitivity and BMI/duration of illness was identified by some authors [27]. Moreover, white matter abnormalities seem to characterize the early stages of the disorder [28], while BMI was proposed as a clinical marker differentiating peak-onset versus late-onset AN [29]. In addition, substance use disorders appear to be more prevalent in patients with a lower BMI [17] and have been associated with severity of depressive symptoms [30] as well as higher mortality [31].

Despite these findings, so far, few studies have investigated the role of biochemical and clinical parameters in discerning AN severity. In addition, AN severity has been measured using diverse criteria [11,17,32], leading to non-homogeneous pieces of evidence. Thus, findings on the topic are preliminary without the possibility to draw definitive conclusions [16]. Carrying out this study, we hypothesized that more severe forms of AN may be characterized by specific biochemical alterations, or may present peculiar clinical features, which might represent potential markers of severe AN. Of note, the identification of biological and clinical markers of severity would allow early and more targeted management of AN patients in the framework of precision medicine. In light of these considerations, the objective of the present research was to investigate the potential association of some biological and clinical factors with BMI, considered to be an indicator of AN severity. BMI was analyzed as a continuous variable, considering AN severity as a spectrum, in order to avoid categorizations and to make our investigation more adherent to the variability of the clinical manifestations of AN.

## 2. Materials and Methods

### 2.1. Sample and Study Design

This was a cross-sectional study. The study was approved by the Ethics Committee of San Gerardo Hospital (4060—approval 20/03/2023), and the research project complied with the Helsinki Declaration of 1975, as revised in 2008, regarding medical research in humans in line with good clinical practice requirements.

Inclusion criteria were: (1) diagnosis of AN according to the Diagnostic and Statistical Manual of Mental Disorders (DSM) 5th edition (DSM-5) [33]; (2) ability and willingness to provide informed consent; (3) age included between 17 and 60 years. Exclusion criteria were: (1) intellectual disability; (2) malnourishment due to severe organic disease; (3) pregnancy or breastfeeding; (4) taking vitamin B12 supplements; (5) presence of severe acute inflammatory diseases or medical/neurological chronic illness that could significantly affect biochemical parameters.

A total of 78 subjects were recruited over 18 months (from July 2021 to December 2022) from the Inpatient Clinic of Fondazione IRCCS Ca’ Granda, Ospedale Maggiore Policlinico (Milan) and the Outpatient Clinic for Eating Disorders of Fondazione IRCCS San Gerardo dei Tintori (Monza).

### 2.2. Assessment

All patients were evaluated by an expert senior psychiatrist. The diagnosis of AN, as well as that of psychiatric comorbidities, was performed according to DSM-5 criteria and confirmed using the Italian version of the Eating Disorder Examination-EDE-Interview-17.0D [34].

During the first psychiatric evaluation, weight and height were measured, and BMI was calculated using the formula = weight in kg/height in m^2^.

Moreover, the following socio-demographic and clinical variables were collected: age, gender, marital status, occupation, age at onset, duration of illness, duration of untreated illness (DUI), presence and type of substance misuse, poly-substance misuse, presence and type of family history for mental disorders, presence and type of psychiatric comorbidities, presence of multiple psychiatric comorbidities, type of comorbid personality disorder, current psychotherapy, current psychopharmacological treatment, presence of poly-therapy, presence of medical comorbidities, presence of multiple medical comorbidities. DUI was defined as the time period elapsed between AN onset and the first appropriate treatment according to international guidelines [35,36].

### 2.3. Blood Collection

Blood samples of outpatients at Fondazione IRCCS San Gerardo dei Tintori (Monza) were collected fasting in the morning during the first psychiatric visit or the medical assessment at the outpatient clinic.

Blood samples of hospitalized patients at Fondazione IRCCS Ca’ Granda Ospedale Maggiore Policlinico (Milano) were collected between 6.00 a.m. and 8.00 a.m. in fasting condition at the beginning of hospitalization.

Among all biochemical parameters routinely dosed during hospitalization or in our outpatient clinic, we collected plasma levels of the following markers, which were available for more than 30% of the sample: total number of red blood cells (10^12^/L), red cell volume (fL), hemoglobin (g/dL), total number of white blood cells (10^9^/L), lymphocytes (10^9^/L), neutrophils (10^9^/L), platelets (10^9^/L), plasma levels of iron (mcg/dL), folic acid (ng/mL), vitamin D (ng/mL), vitamin B12 (pg/mL), TC (mg/dL). Levels of biochemical parameters were measured by enzyme-linked immunosorbent assay (ELISA) kits. NLR and PLR were calculated by dividing, respectively, the total neutrophils count and the total platelet count by the total lymphocyte count.

### 2.4. Statistical Analyses

Descriptive analyses of the entire sample were performed. Frequencies with percentages were reported for qualitative variables, while mean with standard deviation was calculated for quantitative ones.

BMI (kg/m^2^) was compared among groups defined by qualitative variables using one-way analyses of variance (ANOVAs).

The relationship between BMI (kg/m^2^) and quantitative variables (including biochemical values) was first explored using Pearson’s correlations; then, the statistically significant variables were inserted in linear regression models as independent variables, and BMI was considered the dependent variable.

Statistical analyses were performed through The Statistical Package for Social Sciences (SPSS) for Windows (version 28.0). The level of statistical significance was set at *p* ≤ 0.05.

## 3. Results

### 3.1. Descriptive Analyses

A total of 78 patients were included. Among them, 12 (15.4%) were hospitalized, and 66 (84.6%) were outpatients. The mean age of the whole sample was 23.19 (±8.32) years. Mean BMI was 17.21 (±2.21) (kg/m^2^). Most subjects were female (*n* = 77, 98.7%), did not present substance misuse (*n* = 67, 88.2%), and had no medical comorbidities (*n* = 55, 72.4%). In patients with a medical comorbidity (*n* = 21, 27.6%), the most frequent conditions were anemia or endocrine diseases.

The results from descriptive and correlation analyses, as well as the BMI values, are reported in Table 1 for qualitative variables and in Table 2 for quantitative variables.

According to the DSM-5 classification of severity, 38 patients (51.3%) were affected by mild AN, 17 (23.0%) by moderate AN, 12 (16.2%) by severe AN, and 7 (9.5%) by extremely severe forms of AN. Of the total sample, 28 (35.9%) presented the binge-eating/purging type, and 50 (64.1%) the restrictive type of AN.

With regard to the main pharmacological treatment at the first psychiatric assessment or at the time of hospitalization, data were available for 58 patients: 45 were not currently in treatment, while the others were taking different pharmacological treatments: specifically, 1 was treated with setraline, 2 with escitalopram, 1 with mirtazapine, 2 with duloxetine, 1 with olanzapine, 2 with quetiapine, 1 with aripiprazole, 2 with asenapine, and 1 with haloperidol.

### 3.2. One-Way ANOVAs

BMI was significantly lower in hospitalized patients compared to outpatients (F = 4.662; *p* = 0.034) and in subjects under pharmacological treatment (F = 5.733; *p* = 0.019) or poly-therapy (F = 5.635; *p* = 0.021).

### 3.3. Linear Regression Analyses

All models that considered quantitative parameters (including peripheral markers) as independent variables and BMI as dependent variables were reliable (Durbin-Watson’s tests: >1.5 and <2.5).

**Figure 1:**
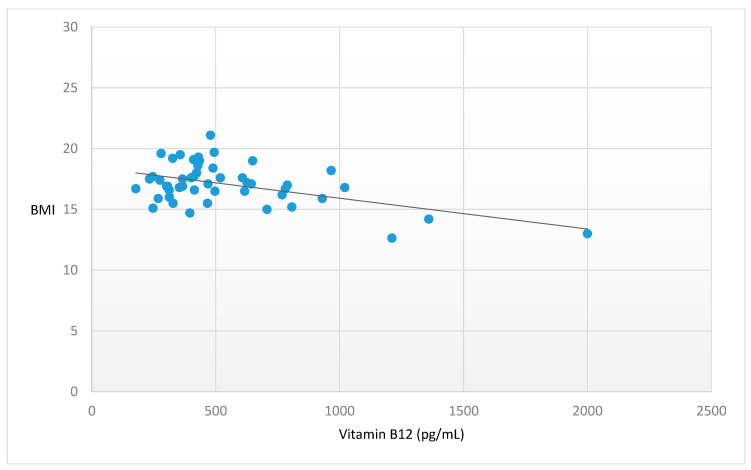
Significant association between Vitamin B12 and BMI. Legend: BMI = Body Mass Index. Statistics: β = −0.556, t = −4.055, *p* ≤ 0.001.

**Figure 2:**
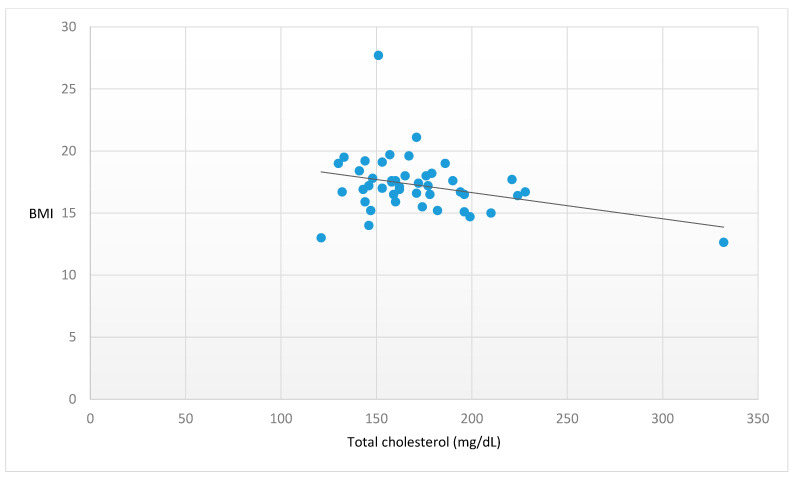
Significant association between total cholesterol and BMI. Legend: BMI = Body Mass Index. Statistics: β = −0.320, t = −2.331, *p* = 0.027.

**Figure 3:**
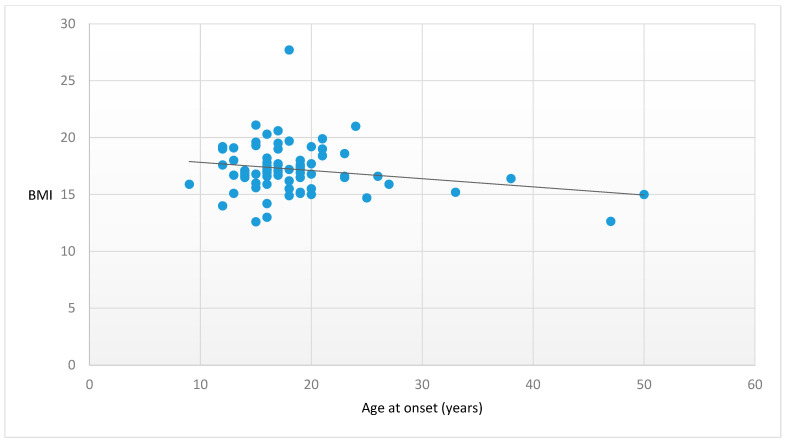
Association between age at onset and BMI. Legend: BMI = Body Mass Index. Statistics: β = −0.376, t = −2.033, *p* = 0.058.

Higher vitamin B12 (β = −0.556, *p* < 0.001) (Figure 1) and higher TC (β = −0.320, *p* = 0.027) (Figure 2) plasma levels were significantly associated with lower BMI. Moreover, later age at onset showed a trend towards statistical significance in the association with a lower BMI (β = −0.376, *p* = 0.058) (Figure 3). No further significant associations were found between the other peripheral parameters and BMI (*p* > 0.05).

## 4. Discussion

The present study sought to explore the relationship between AN severity, as measured by BMI, and several clinical/biochemical factors in order to identify potential markers of disease severity. Our results indicated that patients with more severe forms of anorexia had higher plasma concentrations of TC and vitamin B12. In addition, current pharmacological treatment, the presence of poly-therapy, and an inpatient setting were factors associated with a lower BMI. Finally, a later age at onset was associated with a lower BMI, with a trend towards statistical significance.

Although higher levels of TC in AN patients compared to controls have been repeatedly reported [15], fewer studies have investigated the association between cholesterol plasma levels and the severity of the disorder. In line with our findings, Speranza and colleagues [18] found higher TC levels in subjects with a lower BMI, although statistical significance was not achieved. Similarly, in an older study [37], concentrations of low-density lipoprotein (LDL) and TC were negatively associated with BMI in acutely ill individuals (i.e., prior to partial weight restoration). A recent meta-analysis also found a negative significant association between BMI and very-low-density lipoprotein (VLDL) in acutely ill AN patients, although the result was deemed exploratory due to the small number of included studies [14].

Cholesterol levels in AN patients usually fall within the reference range for healthy subjects, although concentrations above the upper limit have also been documented [38,39]. The paradoxical increase of TC in AN has been identified since the 1960s [40,41], and some reports exist of patients with high TC levels developing myocardial infarction [39,42]. Nonetheless, ischemic heart disease and overall cardiovascular risk do not seem to be more common in AN than in the general population [43,44]. Based on several guidelines, such as the National Cholesterol Education Program [45], there is currently no indication for lipid-lowering pharmacological treatment in AN—due to lipid concentrations, the typical age of the patients, their predominant gender, their protective HDL levels, and the lack of concomitant cardiovascular risk factors [43]. In addition, since treatment is not indicated, measuring cholesterol as part of standard biochemical evaluation is not advisable [44].

Several hypotheses have been proposed to explain high TC in AN patients, such as a reduction in cholesterol metabolism resulting from malnutrition-induced decreases in T3 secretion [46]. Additionally, a role for the gut microbiome in altering cholesterol absorption has also been posited [47,48]. It remains to be elucidated whether higher cholesterol levels in individuals with a lower BMI is merely the result of a greater degree of food deprivation/starvation (state effect) [49] or whether there is a more fundamental lipid dysregulation (trait effect) that may worsen in more severe forms of the disorder. The current literature seems to point towards a global lipid dysregulation in AN, sustained by a complex interplay between liver function, lipid metabolism, and hormones (e.g., insulin, cortisol, estradiol, growth hormone, and triiodothyronine) [50,51], as most studies showed persistently elevated lipid concentrations after weight restoration [52,53,54]. In addition, several authors pointed out an inverse relation between total cholesterol levels and vitamin D [55,56], which plays an important role in mood regulation by favoring the release of dopamine in the central nervous system [57]. Future research should verify if the severity of anorexia predisposes to more frequent comorbidity with major depression, as indicated by some authors [58].

Emerging evidence from Duncan et al. [59] indicates a direct genetic correlation between AN and HDL cholesterol, suggesting that increased cholesterol concentrations in AN may not be solely justified by the effects of starvation but more probably arise from a common genetic underpinning. Of note, the potential role of cholesterol in the etiopathogenesis of mental disorders has already been highlighted [35,60]. Further longitudinal studies should explore changes over time in BMI and metabolic profiles in AN in order to elucidate the potential involvement of cholesterol in the pathogenesis of severe forms of AN.

Along with cholesterol, serum vitamin B12 levels showed a negative association with BMI. Despite persistent dietary restriction, normal or even elevated levels of vitamin B12 have been reported in AN [11,12,61], in line with our findings (mean 529.43 +/− 325.68 pg/mL). With vitamin B12 stored in the liver [62], this seemingly paradoxical finding is most likely explained by the leakage of the vitamin into the bloodstream as a result of hepatic dysfunction [63,64,65]. In line with this hypothesis, two studies found a positive association between vitamin B12 parameters and hyper-transaminemia in acute AN patients [11,12]. Of note, elevations of liver enzymes have been previously associated with lower BMI in AN patients [66,67]. Moreover, Corbetta et al. [11] found that higher cobalamin levels correlated with higher Eating Disorder Inventory (EDI) scores, suggesting that vitamin B12 might represent not only an early marker of liver damage but a broader indicator of AN severity. Consistently, a previous study reported higher vitamin B12 levels in a subgroup of patients with BMI < 17 [17]. It should, however, be noted that contrary to these findings, some severely malnourished AN patients also display cobalamin deficits [8]. Hence, the validity of vitamin B12 as a marker of illness severity in AN requires further investigation. Understanding the impact of vitamin B12 alterations on cardiovascular risk would also appear crucial. Although, to our knowledge, this has not been specifically studied in AN patients, both low (<369.1 pg/mL) and high (≥506.1 pg/mL) cobalamin levels have been associated with a higher risk of mortality due to cardiovascular disease (CVD) in persons with type 2 diabetes, as well as in the general population [68,69,70]. Interestingly, while vitamin B12 supplementation may reduce the risk of mortality [71] and stroke [72] by lowering circulating homocysteine, elevated vitamin B12 levels seem to predispose to a higher risk of CVD mortality independently of homocysteine levels [68]. Future studies should clarify the effect of cobalamin alterations on the cardiovascular health of anorexic patients.

Pharmacotherapy does not typically represent the first-line treatment for AN [73]. However, several patients receive adjunctive pharmacological treatments, especially those who do not respond to psychotherapy or nutritional rehabilitation [74]. The literature suggests that more severe patients may be more likely to present non-eating-related symptoms, such as anxiety and depressed mood [75,76], and that depressive, anxious, and obsessive symptoms tend to be more elevated with a lower BMI [77,78,79]. Moreover, low BMI was identified as a key predictor of poor treatment response in inpatients receiving multimodal, non-pharmacological therapy [80]. In this framework, our findings of a lower BMI in patients taking pharmacological treatment suggest that more severe forms of the disorder are likely to benefit from the augmentation of psychotherapy with pharmacotherapy after failure to respond to first-line treatment strategies. In addition, the control of several non-eating-related symptoms might require the use of multiple medications, in line with our results showing an association between greater AN severity and poly-therapy. It is worth noting that medication use might be associated with an increase in BMI in AN [81,82,83]. This would appear in contrast with our result of a lower BMI in patients under pharmacological treatment. However, medications might have been prescribed shortly before recruitment, so BMI improvements might not have become apparent yet. Indeed, studies documenting weight gain were carried out throughout a treatment period of at least 3 weeks [82,83,84]. Moreover, as our data refer to the first presentation at our inpatient or outpatient services, we have no information on treatment adherence.

Our finding of a lower BMI in inpatient settings could be easily explained by the greater severity of the disorder, which requires inpatient management. Indeed, guidelines recommend intensive medical assistance for patients with a BMI < 15 [36].

Finally, in our study, later age at onset showed a correlation with greater AN severity, with a trend towards significance. Different symptoms, ascribable to other clinically relevant disorders, may precede the onset of AN, suggesting the presence of comorbidities that may sustain more complicated forms of AN. This is in line with previous reports of a lower weight and lower BMI in a subgroup of patients with late illness onset [29,85]. In addition, a later age at onset was associated with poor outcomes in a large clinical longitudinal study with a very long-term follow-up [86].

### Limitations

The results of the present study should be interpreted carefully in light of several limitations. First, the sample size was relatively small, leading to a potential reduction of the power of statistical analyses. Second, peripheral markers were chosen a priori, according to routinely assessed panels, which do not include, for example, other vitamins or cytokine plasma levels. In addition, we only measured TC, leaving out the complete lipid panel and a control group is missing for comparison on these parameters. Moreover, only total vitamin B12 was quantified, while other measures were useful markers of cobalamin status, such as holotranscobalamin [87,88] or a combination of multiple biomarkers reflecting the metabolic function of the vitamin [89,90]. We are also planning to expand the present study measuring circulating levels of Tumor Necrosis Factor-α, IL-6, and C reactive protein, as well as other inflammatory and antioxidant factors. Third, we measured AN severity using BMI as a single parameter, according to the DSM-5 classification [33]. However, the severity of AN is a more complex concept, including, for example, the rapidity of weight loss and body composition, as well as peculiar psychological constructs, such as body image distortion [91], measured by specific psychometric scales. Fourth, 37.2% of the subjects were taking pharmacological treatment; about half of them were treated with antipsychotics, and the other 50% with antidepressants. Although evidence regarding the clinical benefit of these molecules for weight restoration in AN is limited and contrasting [92], their efficacy on comorbid anxiety, obsessive thoughts, and depressive symptoms is plausible [93]. Nevertheless, it is noteworthy that antipsychotics, as well as antidepressant drugs, may significantly impact weight and metabolism as a side effect, also affecting and potentially increasing the cardiovascular risk of AN patients [44]. In this sense, pharmacotherapy may represent a bias in analyzing metabolic markers such as total cholesterol in AN subjects. Fifth, despite the exclusion of patients taking supplements of vitamin B12, some other supplements, as well as substance misuse, might have influenced the values of some biochemical markers, thus representing, together with the duration of pharmacological treatment, potential confounding factors.

Taken together, all these aspects may have limited the reliability of our results. Moreover, the cross-sectional design of the study may imply the presence of both selection and information bias, as the potential recall bias for self-reported information (e.g., age at onset) may influence and limit the generalization of our findings.

In conclusion, our study demonstrated that total cholesterol and vitamin B12 plasma levels are increased in more severe forms of AN, although their role as potential biomarkers of severity should be explored in more detail. Some clinical factors, such as being older at onset, taking pharmacological treatment, and poly-therapy, characterized AN patients with lower BMI. In clinical practice, subjects affected by “late-onset” AN or taking pharmacological treatment or poly-therapy, as well as subjects with higher vitamin B12 or total cholesterol plasma levels, may deserve more attention in light of the potential greater severity of clinical manifestations. Nevertheless, due to the cross-sectional design of our study and the limitations of our research, a definitive conclusion cannot be drawn. Further longitudinal studies on larger samples, considering both anthropometric and psychological parameters of AN severity, are needed to identify potential predictive factors of greater severity in AN, which may help to optimize preventive and therapeutic strategies and to improve the course and outcome of this disabling disorder.

## Figures and Tables

**Table 1:** Socio-demographic and clinical qualitative variables of the total sample (N = 78).

Variables		N (%)	BMI (kg/m^2^) (Mean ± SD)	F *	*p*
**Setting**	Inpatients	12 (15.4%)	15.7 ± 2.21	4.66	**0.03**
	Outpatients	66 (84.6%)	17.4 ± 2.15		
**Sex**	Male	1 (1.3%)	16.8 ± 0.0	0.03	0.85
	Female	77 (98.7%)	17.2 ± 2.22		
**Being in a relationship**	Yes	20 (29.9%)	16.9 ± 1.83	1.37	0.25
	No	47 (70.1%)	17.6 ± 2.37		
	Missing	11			
**Occupational status**	Student	50 (67%)	17.4 ± 2.31	0.28	0.76
	Unemployed	13 (17%)	16.9 ± 2.48		
	Worker	12 (16%)	17.1 ± 1.65		
	Missing	3			
**Substance misuse**	Yes	9 (11.8%)	16.3 ± 1.81	1.77	0.19
	No	67 (88.2%)	17.4 ± 2.25		
	Missing	2			
**Main substance of misuse**	None	67 (89.3%)	17.4 ± 2.25	0.75	0.53
	Alcohol	3 (4.0%)	16.8 ± 3.03		
	Cannabinoids	4 (5.3%)	15.7 ± 0.93		
	Others	1 (1.4%)	15.5 ± 0.0		
	Missing	3			
**Poly-substance misuse**	Yes	3 (4.0%)	16.0 ± 0.71	0.63	0.43
	No	72 (96.0%)	17.3 ± 2.26		
	Missing	3			
**Family history of mental disorders**	Yes	27 (35.5%)	17.0 ± 2.64	0.45	0.50
	No	49 (64.5%)	17.4 ± 1.95		
	Missing	2			
**Type of family history for mental disorders**	None	49 (64.5%)	17.4 ± 1.95	0.79	0.58
	Anxiety disorders	4 (5.3%)	17.7 ± 1.29		
	Bipolar disorder	1 (1.3%)	17.1 ± 0.0		
	MDD	12 (15.8%)	17.6 ± 3.59		
	Eating disorders	8 (10.5%)	16.4 ± 1.36		
	SUD	1 (1.3%)	14.0 ± 0.0		
	Others	1 (1.3%)	15.2 ± 0.0		
	Missing	2			
**Psychiatric comorbidity**	Yes	27 (34.6%)	17.6 ± 2.72	1.13	0.29
	No	51 (65.4%)	17.0 ± 1.85		
**Type of psychiatric comorbidity**	None	51 (65.4%)	17.0 ± 1.85	1.45	0.22
	Anxiety disorder	8 (10.3%)	19.1 ± 3.57		
	MDD	11 (14.1%)	16.8 ± 2.54		
	OCD	3 (3.8%)	17.4 ± 0.51		
	Personality disorder	4 (5.1%)	16.7 ± 1.76		
	Others	1 (1.3%)	16.9 ± 0.0		
**Presence of multiple psychiatric comorbidities**	Yes	8 (10.3%)	15.8 ± 2.52	2.07	0.16
	No	70 (89.7%)	17.3 ± 2.17		
**Type of comorbid personality disorder**	None	65 (89.0%)	17.4 ± 2.21	1.31	0.28
	Borderline	4 (5.5%)	15.2 ± 1.77		
	Schizoid	1 (1.4%)	16.9 ± 0.0		
	Schizotypal	2 (2.7%)	14.6 ± 2.8		
	NOS	1 (1.4%)	19.0 ± 0.0		
	Missing	5			
**Current psychotherapy**	Yes	4 (6.5%)	15.5 ± 1.94	2.80	0.10
	No	58 (93.5%)	17.5 ± 2.29		
	Missing	16			
**Current pharmacological treatment**	Yes	29 (37.2%)	16.2 ± 1.75	5.73	**0.02**
	No	49 (62.8%)	17.5 ± 2.31		
**Presence of poly-therapy**	Yes	12 (19.4%)	15.7 ± 2.21	5.64	**0.02**
	No	50 (80.6%)	17.7 ± 2.22		
	Missing	16			
**Medical comorbidity**	Yes	21 (27.6%)	17.3 ± 1.73	0.00	0.97
	No	55 (72.4%)	17.2 ± 2.42		
	Missing	2			
**Medical poly-comorbidity**	Yes	3 (4.1%)	16.9 ± 2.65	0.08	0.78
	No	71 (95.9%)	17.3 ± 2.23		
	Missing	4			

Legend: bold = statistically significant values; * resulting from one-way analyses of variance.

**Table 2:** Descriptive analyses and values of BMI/Pearson’s correlation according to quantitative variables of the total sample (N = 78).

Variables	Mean ± SD	Pearson’s Correlation (r)	*p*
Age (years)	23.2 ± 8.32	−0.281	**0.015**
Age at onset (years)	18.6 ± 6.61	−0.22	0.06
DUI (years)	2.1 ± 2.69	−0.104	0.384
Duration of illness (years)	4.1 ± 5.77	−0.143	0.228
Red Cells (10^12^/L)	4.3 ± 0.44	0.173	0.442
(n.v. 4.50–6.00 × 10^12^/L)			
Red cell volume (fL)	90.8 ± 5.47	0.276	0.214
(n.v. 80.0–99.0 fL)			
Hemoglobin (g/dL)	13.0 ± 1.26	0.232	0.299
(n.v. 14.0–18.0 g/dL)			
White cells (10^9^/L)	5.7 ± 1.74	0.165	0.166
(n.v. 4.00–11.00 × 10^9^/L)			
Lymphocytes (10^9^/L)	2.1 ± 0.74	−0.013	0.916
(n.v. 1.00–5.00 × 10^9^/L)			
Neutrophils (10^9^/L)	3.2 ± 1.67	0.03	0.84
(n.v. 1.50–7.50 × 10^9^/L)			
Platelets (10^9^/L)	228.2 ± 49.01	0.071	0.551
(n.v. 140.0–440.0 × 10^9^/L)			
NLR	1.5 ± 0.92	0.09	0.539
(n.v. 1–2)			
PLR	117.5 ± 41.47	0.051	0.675
(n.v. 90–210)			
Iron (mcg/dL)	106.6 ± 74.72	−0.005	0.971
(n.v. 65.0–178.0 mcg/dL)			
Vitamin D (ng/mL)	30.4 ± 14.14	−0.061	0.714
(n.v. 20.0–40.0 ng/mL)			
Folate (ng/mL)	8.9 ± 6.24	−0.247	0.081
(n.v. 2–20 ng/mL)			
Vitamin B12 (pg/mL)	529.4 ± 325.68	**−0.499**	**<0.001**
(n.v. 200.0–900.0 pg/mL)			
Cholesterol (mg/dL)	172.3 ± 34.60	**−0.315**	**0.035**
(n.v. < 190 mg/dL)			

Legend: n.v. = normal ranges detected in healthy adult populations. Bold = statistically significant values.

## Data Availability

The datasets generated during and/or analyzed during the current study are available from the corresponding author upon reasonable request.
